# 550. Increase in acute rheumatic fever and rheumatic heart disease in Alaska, 2022–2025

**DOI:** 10.1093/ofid/ofaf695.023

**Published:** 2026-01-11

**Authors:** Joe B B Silva, Victoria Balta, Minal Ahson, Veena Ramachandran, Sara Bressler, Andra Rasmussen, Heather Wheelock, Rosalyn Singleton, Kristina Morris, Adrienne Kishi, David Bridgman-Packer, James Keck, Christopher Gregory, Louisa Castrodale, Joe McLaughlin, Heather Scobie

**Affiliations:** CDC, Atlanta, Georgia; CDC, Atlanta, Georgia; CDC, Atlanta, Georgia; Centers for Disease Control and Prevention, Atlanta, GA; CDC, Atlanta, Georgia; CDC, Atlanta, Georgia; CDC, Atlanta, Georgia; Alaska Native Tribal Health Consortium, Anchorage, Alaska; Yukon-Kuskokwim Health Corporation, Bethel, Alaska; Yukon-Kuskokwim Health Corporation, Bethel, Alaska; Southcentral Foundation, Anchorage, Alaska; Alaska Native Tribal Health Consortium, Anchorage, Alaska; Centers for Disease Control and Prevention, Atlanta, GA; Section of Epidemiology, Division of Health and Social Services, State of Alaska, Anchorage, AK; Alaska Division of Public Health, Anchorage, Alaska; CDC, Atlanta, Georgia

## Abstract

**Background:**

In February 2025, pediatricians alerted the Alaska Department of Health to a possible increase in cases of acute rheumatic fever (ARF) and rheumatic heart disease (RHD). ARF/RHD are immune-mediated sequelae of group A *Streptococcus* (GAS) infections. ARF primarily affects school-aged children and can progress to RHD without appropriate antibiotic treatment and prophylaxis. RHD is characterized by damage to heart valves and can result in death. Early diagnosis, appropriate antibiotic treatment, and prophylaxis for people with ARF/RHD reduce the risk of ARF recurrence and RHD progression.

**Table 1:**
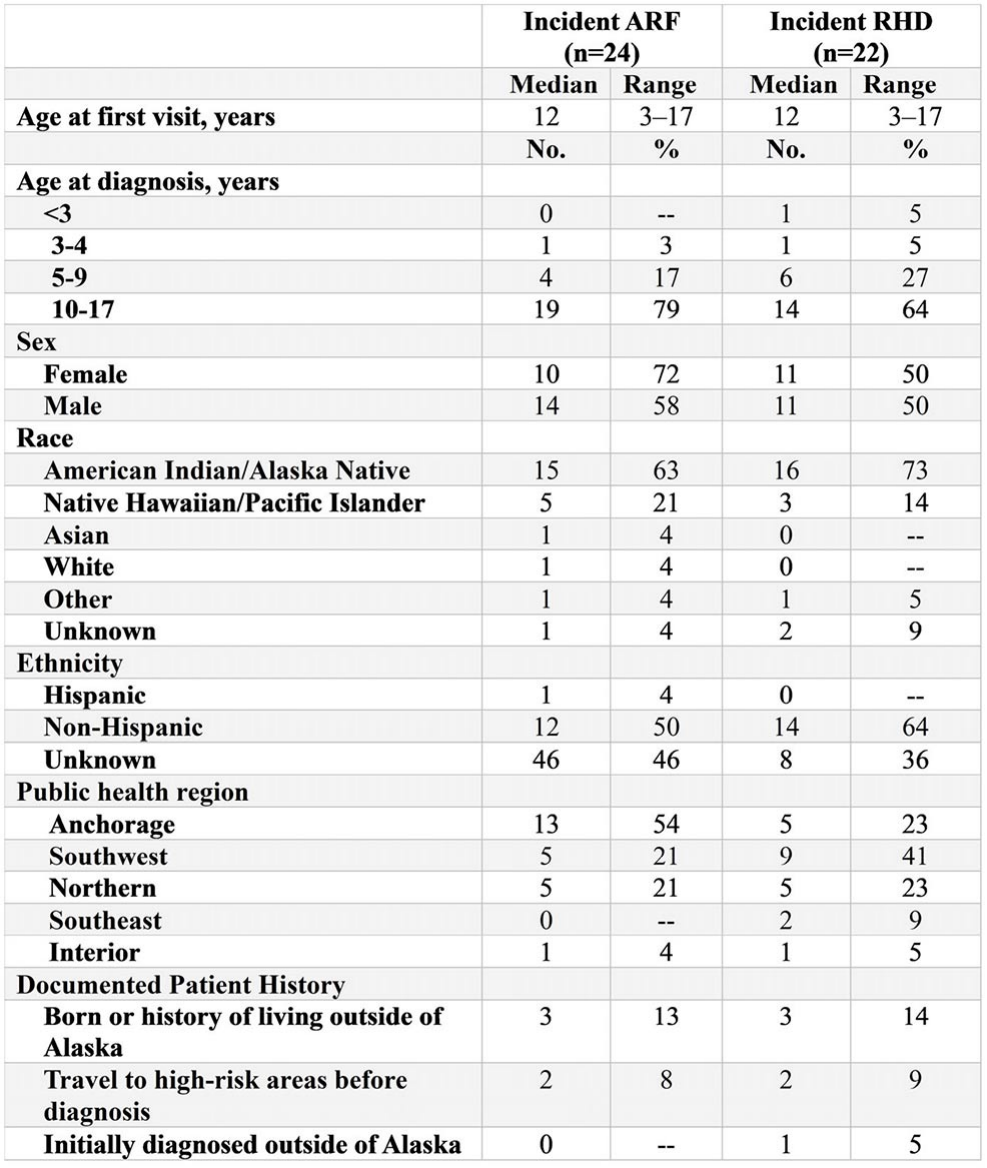
Demographic characteristics of patients with ARF and RHD included in medical records review, Alaska, Jan 2022–Feb 2025. (N=52)

**Table 2.**
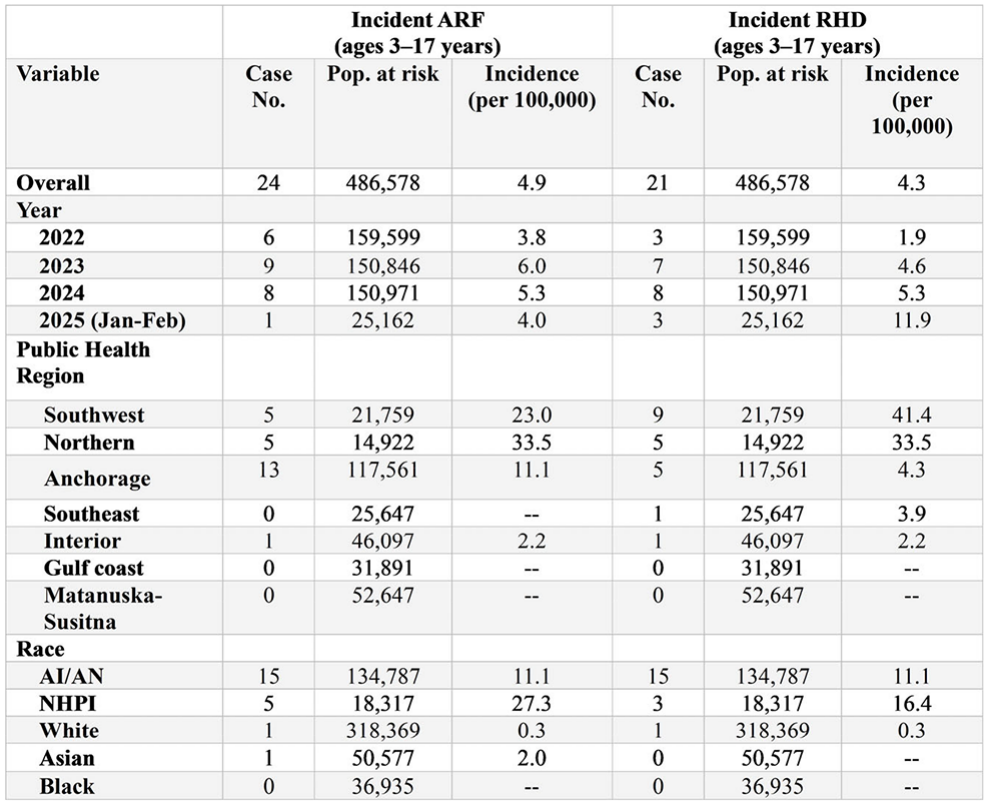
Incidence of ARF/RHD among ages 3-17 years from medical records review, Alaska, Jan 2022–Feb 2025.

**Methods:**

We calculated ARF/RHD incidence among children aged 3–17 years living in Alaska during January 2022–February 2025 using data reported by healthcare systems in response to a statewide call for cases. We conducted medical chart reviews to collect information on presenting signs and symptoms, prior GAS infections, clinical management, and outcomes. We also conducted a secondary data analysis of the Indian Health Service’s (IHS) National Patient Information Reporting System to calculate incidence of ARF/RHD during 2014–2023 among Alaska Native children aged 3–17 who received care within the Tribal Health System.

**Figure 1.**
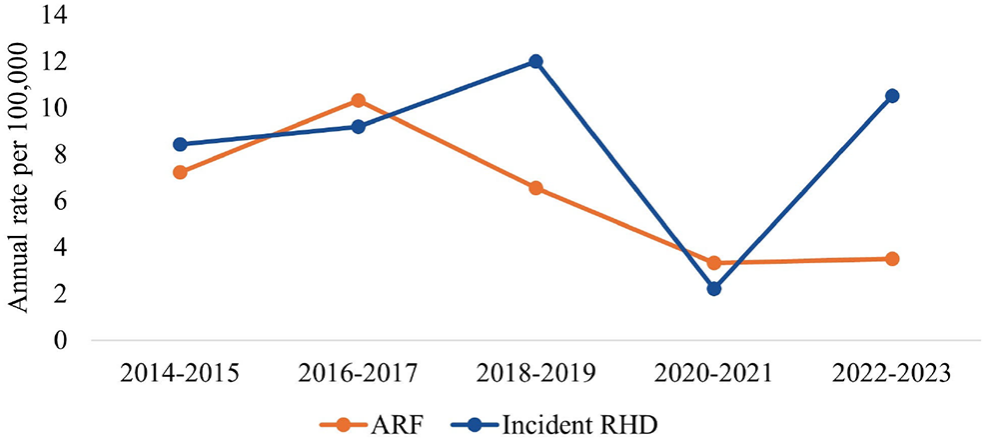
Indian Health Service National Patient Information Reporting System trends in reported incidence of new ARF and RHD among AI/AN children aged 3–17 years, Alaska, 2014–2023

**Figure 2.**
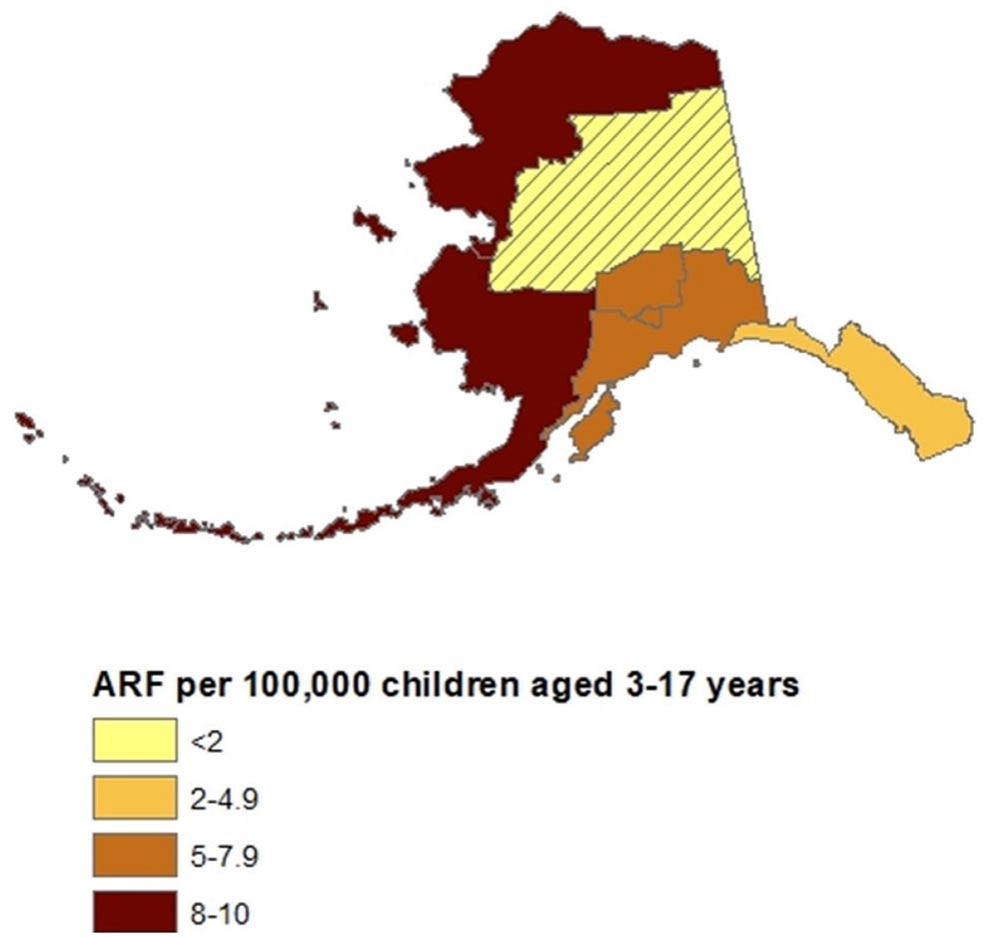
Indian Health Service’s National Patient Information Reporting System reported average ARF incidence (3–17 years) among AI/AN people by region, Alaska, 2014–2023

**Results:**

ARF and RHD incidence among children aged 3–17 increased from 3.8 and 1.9 per 100,000 persons per year in 2022 to 5.3 and 5.3 per 100,000 persons per year, respectively, in 2024. Incidence was highest in the northern (ARF: 33.5; RHD: 33.5) and southwestern (ARF: 23.0; RHD: 41.4) regions of Alaska and among children who were Alaska Native (ARF: 11.1; RHD: 11.1) or Native Hawaiian/Pacific Islander (ARF: 27.3; RHD: 16.4). Analysis of IHS data found the average annual incidences of ARF/RHD during 2014–2023 among Alaska Native children aged 3–17 were 6.2 per 100,000 persons and 8.5 per 100,000 persons, respectively.

**Conclusion:**

ARF/RHD incidence in Alaska is higher than the rest of the United States, particularly among Alaska Native and Native Hawaiian/Pacific Islander people. The observed recent increases may be linked to factors such as a rise in GAS infections. Potential opportunities to improve early diagnosis and clinical management of GAS infections and sequelae can inform public health recommendations and clinical guidance.

**Disclosures:**

All Authors: No reported disclosures

